# Process of the Functional Reorganization of the Cortical Centers for Movement in GBM Patients: fMRI Study

**DOI:** 10.1007/s00062-015-0398-7

**Published:** 2015-05-19

**Authors:** A. Majos, B. Bryszewski, K. N. Kośla, L. Pfaifer, D. Jaskólski, L. Stefańczyk

**Affiliations:** 1 Department of Radiology and Diagnostic Imaging, Medical University of Łódź, Kopcińskiego 22, 90-153 Łódź, Poland; 2 Department of Neurosurgery, Medical University of Łódź, Kopcińskiego 22, 90-153 Łódź, Poland

**Keywords:** Brain plasticity, fMRI, Brain tumor, Movement centers

## Abstract

**Purpose:**

The aim of this study was to verify whether the functional reorganization of motor cortex is associated with the increase in the size of WHO type IV glioma lesion, that is, disease duration and development, and whether surgical treatment has an impact on cerebral plasticity.

**Methods:**

The study included 16 patients with primary tumors of the brain located at the region of central sulcus. The clinical status of patients and tumor volume was determined. Functional magnetic resonance imaging examinations were performed before and 3 months after operation.

**Results:**

The activity of all cortical centers, both contralateral and ipsilateral, was observed in a group of small as well as large tumors. The intensity of activation and the number of activated clusters of small tumors were almost always higher as compared with the large tumors. The frequency of the activity of contralateral areas was similar during the first and the second examination. In the case of ipsilateral centers, the frequency of activation during the second examination was lower. Mean values of t-statistics during the first examination were higher than during the second examination. Supplementary motor area (SMAa) was the only center for which the mean values of activation intensity remained similar.

**Conclusions:**

SMAa seems to play the most important role in the processes of motor cortex plasticity in high-grade glioma patients. Surgery seems not having a significant influence on the pattern of functional reorganization of the cortical centers for movement. Identification of the individual patterns of the reorganization of motor centers plays an important role in clinical practice.

## Introduction

As it is widely known, the nervous system is not a static structure. The ability to learn, adaptation to new environmental conditions, and the restoration of neural function following brain injuries represent examples of the plasticity of the nervous system. Various pathological conditions of the brain, for example proliferative processes, congenital malformations, stroke, or neurodegenerative disorders, are associated with changes in the location and size of centers controlling various neural functions, involvement of secondary and accessory centers, and cooperation of centers belonging to different systems [1, 2].

Established knowledge of the possible patterns of functional reorganization has the potential to become crucial for clinical practice, including neurosurgery. The primary objective of the surgical approach to brain tumors is the optimal resection of the proliferative lesion; the extent of the resection is strongly correlated with patient’s survival. Information on the actual location of active cortical centers responsible for the control of important vital functions affected by the proliferative process of the brain, as well as on the possibility of compensating for the function of the affected center by intact cortical areas would be crucial in the decision-making process in terms of planning and executing the surgical treatment [3, 4].

High-grade gliomas (HGGs) described within WHO classification represent a particularly challenging group with regards to effective therapy. The prognosis in HGG is poor and, notably, has remained unchanged throughout the last 2 decades. Despite the application of combined therapy, mean duration of survival of HGG patients is 12 months [5, 6].

The aim of this study was to verify whether the functional reorganization of motor cortex is associated with the increase in the size of WHO type IV glioma lesions, that is, disease duration and development, and whether surgical treatment has an impact on cerebral plasticity.

## Methods

The study included 16 patients with primary supratentorial tumors of the brain located at the region of central sulcus, among them 11 women and 5 men. Mean age of the patients was 53.1 ± 13.1 years; individuals younger than 56 years of age comprised one half of the group.

Clinical and neurological examination was conducted at the Clinic of Neurosurgery and Oncology of Central Nervous System, while magnetic resonance (MR) imaging took place at the Department of Radiology and Imaging. The study was approved by the local ethics committee (decision no. RNN/123/09/KE) and conducted in accordance with the principles established by the Helsinki Convention. Informed consent was obtained from all the participants.

The study employed the following inclusion criteria: (1) tumor located in the region of the central sulcus, no further than 15 mm from the sulcus, (2) lack of previous neurosurgical procedures, (3) patient dexterity, (4) general and neurological status enabling functional magnetic resonance imaging (fMRI), (5) consent for neurosurgical treatment, and (6) postoperative histopathological diagnosis of WHO grade IV neuroepithelial tumor.

A total of 14 patients (*F* = 0.87) had lesions within the left hemisphere, and 2 (*F* = 0.13) within the right hemisphere. The location of the lesions with respect to the central sulcus was as follows:

- anteriorly to the sulcus—glioma involving premotor/supplementary motor area (SMAa) and part of the primary motor area—eight cases (*F* = 0.50),
- directly within the cortex of the primary motor area—two cases (*F* = 0.13), and
- posteriorly to the post-central sulcus—tumor involving part of the primary motor area—six cases (*F* = 0.38).

Tumor volume was determined on the basis of imaging; based on this criterion (40 ccm^3^ cut-off value), the patients were divided into those with small or large tumors. The extent of resection was determined on the basis of intraoperative macroscopic examination and control MR performed 3 months post-surgery. The extent of resection was classified as follows: maximal surgical resection corresponded to the removal of 90–100 % of the primary tumor as confirmed on control MR, subtotal resection to 75–90 % removal, and partial resection to < 70 % removal; resection was considered unfeasible if solely a biopsy specimen for histopathological examination was obtained.

The clinical status of patients was defined using the Karnofsky Performance Status (KPS) scale, and the neurological degree of upper limb paresis was determined with the Lovett scale (Lo scale). The presence of seizure episodes was also assessed.

All patients were subjected to surgical treatment with intraoperative electrophysiological monitoring, that is, mapping of cerebral cortex with methods involving motor evoked potentials and sensory evoked potentials.

MR examinations were performed using a 1.5 T scanner, Siemens Magnetom Avanto, Syngo ver. N4VB13A with 12ch HeadMatrix coil (Siemens, Germany). EPI sequences, as well as T1 spin echo were performed using standard parameters routinely used in clinical practice. First, a high resolution T1 anatomical scan was performed with 1 × 1 × 1 mm^3^ isotropic voxels. A three-dimensional FLASH sequence was obtained according to the following protocol: FOV = 256 × 256 mm^2^, matrix = 256 × 256, TR = 8.8 ms, TE = 4.8 ms, FA = 25°, TA = 5’07. Each acquired volume contained 160 slices, 1 mm thick, with no gap. Next, functional scanning was performed with the standard T2* sensitive GRE EPI sequence: TR = 3000 ms, TE = 50 ms, FA = 90 , FOV = 240 × 240 mm^2^, iPAT = 2, pixel bandwidth = 2605 Hz/Px, for thirty-eight 3 mm thick slices with 0.75 mm gap and 3.75 mm in-plane resolution. All slices where positioned in the axial plane parallel to the AC-PC line; 100 volumes were acquired with total TA = 5’12 (4 dummy scans).

All subjects were trained to rehearse a paradigm task for a few minutes before the procedure. Each patient performed a simple finger tapping paradigm. The movement task required the patient to freely move the fingers of the hand contralateral to the hemisphere with the tumor.

### Preprocessing:

Data were analyzed using statistical parametric mapping (SPM5) software (Wellcome Department of Imaging Neuroscience, University College London, London, UK). Movement correction was performed as the first preprocessing step. All patients with movements greater than 2 mm or correlations with stimulating paradigms bigger than 0.5, were excluded from the study. Spatial smoothing of functional images (Gauss smoothing filter with kernel FWHM = 7 mm) was used to compensate for residual distortion of between-subject variability after spatial normalization. A 128-s high pass filter was applied to remove low-frequency fluctuations in the BOLD response.

### Modeling:

General linear modeling was used to establish a model and integrate it with the acquired data. Model timing was derived from individual subject performance logs.

### Statistics:

Hot spot analyses were performed for individual patients. For each voxel, a T-value was calculated. Each voxel with a T-score above the threshold value was treated as an active voxel, with the condition that there were at least four “active voxels” within the nearest vicinity.

The protocol of the study comprised two stages that took place at different time points; the first stage occurred up to 7 days prior to the surgery, and the second 3 months post-surgery. Subjective and physical examination, as well as neurological examination and fMRI were conducted during these stages.

Nine patients were qualified to the second postoperative stage of the study; the remaining seven patients did not satisfy the criteria defined in the protocol.

### The Activity of Cortical Centers:

Contralateral primary motor area (M1a), ipsilateral primary motor cortex (M1u), contralateral premotor area (PMAa), ipsilateral premotor area (PMAu), and SMAa was examined with regards to the frequency of their activation, the intensity of activation (t-statistics), and the size of activated area (number of activated clusters, *k*).

The results were subjected to statistical analysis; due to the small size of the sample the results were not presented as percentage but as a fraction. Relationships between analyzed variables, intergroup differences in the prevalence of various qualitative variables, and the intragroup differences in preoperative and postoperative findings were analyzed with the chi-square test. If the numbers in the four-field table were 0 or 1, the frequencies were compared with the Fisher’s exact test. Parametric tests were not applied as the distributions of most analyzed variables were significantly different from the normal distribution; instead, the nonparametric Mann–Whitney test for two independent samples was used to compare the mean values.

## Results

Histopathological examination revealed 2 gliosarcomas and 14 glioblastomas. There were 6 small tumors, with a volume up to 40 cm^3^, and 10 large tumors with V > 40 cm^3^. Maximal resection was feasible in 5 patients (*F* = 0.31), whereas 11 patients had subtotal resection (*F* = 0.69).

### Clinical Status

Preoperatively, 6 patients were diagnosed with epilepsy (*F* = 0.38), and 10 subjects were free from this condition (*F* = 0.62); after the surgery the presence of seizures was confirmed in 6 individuals (*F* = 0.38), and excluded in another 10 subjects (*F* = 0.62). Therefore, the prevalence of epilepsy prior to and after the surgery was identical (*p* > 0.05, chi^2^ = 0.000). Both prior to and after the surgery, epilepsy was detected in slightly less than every second patient.

The data on the patient’s KPS and the degree of upper limb paresis expressed in the Lovett (Lo) scale is summarized in Table 1. No significant difference was documented with regards to KPS scores determined prior to and after the surgery (*p* > 0.05, chi^2^ = 3.473).

**Table 1:** Clinical status before and after surgery assessed with use of Karnofsky Performance Status (KPS) and the degree of upper limb paresis expressed in the Lovett (Lo) scale

	Before operation		Post operation	
KPS score	*n*	*F*	*n*	*F*
90–100	2	0.12	3	0.19
70–80	9	0.5	3	0.19
≥ 60 < 70	5	0.17	10	0.62
*Lo scale*				
0-go^0^ Lo	6	0.38	3	0.19
1-go^0^ Lo	2	0.12	7	0.44
2-go^0^ Lo	8	0.50	1	0.06
3-go^0^ Lo			5	0.31

In contrast, there was a significant difference in the degree of paresis determined at these two-time points (*p* < 0.01, chi^2^ = 14.222). Following the surgery, a significant decrease in the fraction of patients without paresis was observed (0.19 vs. 0.38 prior to the surgery), along with a significant increase in the fraction of individuals with grade 3 paresis determined in the Lo scale (0.31 vs. 0).

### The Results of fMRI

Aside from primary contralateral motor cortex, which was active in nearly all the patients (*F* = 0.94), activation of SMAa (*F* = 0.75) and PMAa (*F* = 0.50) was also documented on preoperative examination. The ipsilateral centers, PMAu (*F* = 0.25) and M1u (*F* = 0.25), were activated in every fifth patient.

The activity of all cortical centers, both contralateral and ipsilateral, was observed in a group of small as well as large tumors. The frequency of M1a activation was the same in both groups. PMAa and PMAu were more frequently activated in small tumors, while the activation of SMAa and M1u was more frequent in larger tumors.

The intensity of activation and the number of activated clusters of small tumors were always higher as compared with the large tumors, the only exception pertained to PMAa, where both the values of t-statistics and the area of activation were higher in large tumors (Table 2).

**Table 2:** The comparison of frequency of activity (*F*), values of t-statistics (T) and number of clusters (*k*) of primary and secondary areas in patients with tumors of different volume

	V < 40 cm^3^ (*n* = 6)	V > 40 cm^3^ (*n* = 10)
M1a (*n*)	6	9
M1a (*F*)	1.0	0.90
T	15.76 ± 5.60	11.69 ± 5.51
*k*	372.17 ± 106.0	295.22 ± 148.83
V (cm^3^)	24.60 ± 10.62	56.65 ± 17.69
M1u (*n*)	1	3
M1u (*F*)	0.167	0.30
T	13.41 ± 0	5.99 ± 1.70
*k*	513.0 ± 0	125.0 ± 106.06
V (cm^3^)	24.68	55.84 ± 7.01
PMAa (*n*)	4	4
PMAa (*F*)	0.667	0.40
T	6.77 ± 1.78	9.91 ± 4.69
*k*	137.25 ± 126.33	283.0 ± 170.25
V (cm^3^)	24.72 ± 10.54	51.94 ± 9.40
PMAu (*n*)	2	2
PMAu (*F*)	0.333	0.20
T	8.19 ± 3.22	5.92 ± 1.41
*k*	237.0 ± 166.88	193.0 ± 76.31
V (cm^3^)	31.29 ± 9.34	49.26 ± 11.12
SMAa (*n*)	4	8
SMAa (*F*)	0.667	0.80
T	9.30 ± 3.48	6.07 ± 1.03
*k*	353.50 ± 121.70	184.63 ± 89.63
V (cm^3^)	30.48 ± 7.05	57.08 ± 18.53

*M1a* contralateral primary motor area, *M1u* ipsilateral primary motor cortex, *PMAa* contralateral premotor area, *PMAu* ipsilateral premotor area, *SMAa* supplementary motor area

**Fig. 1:**
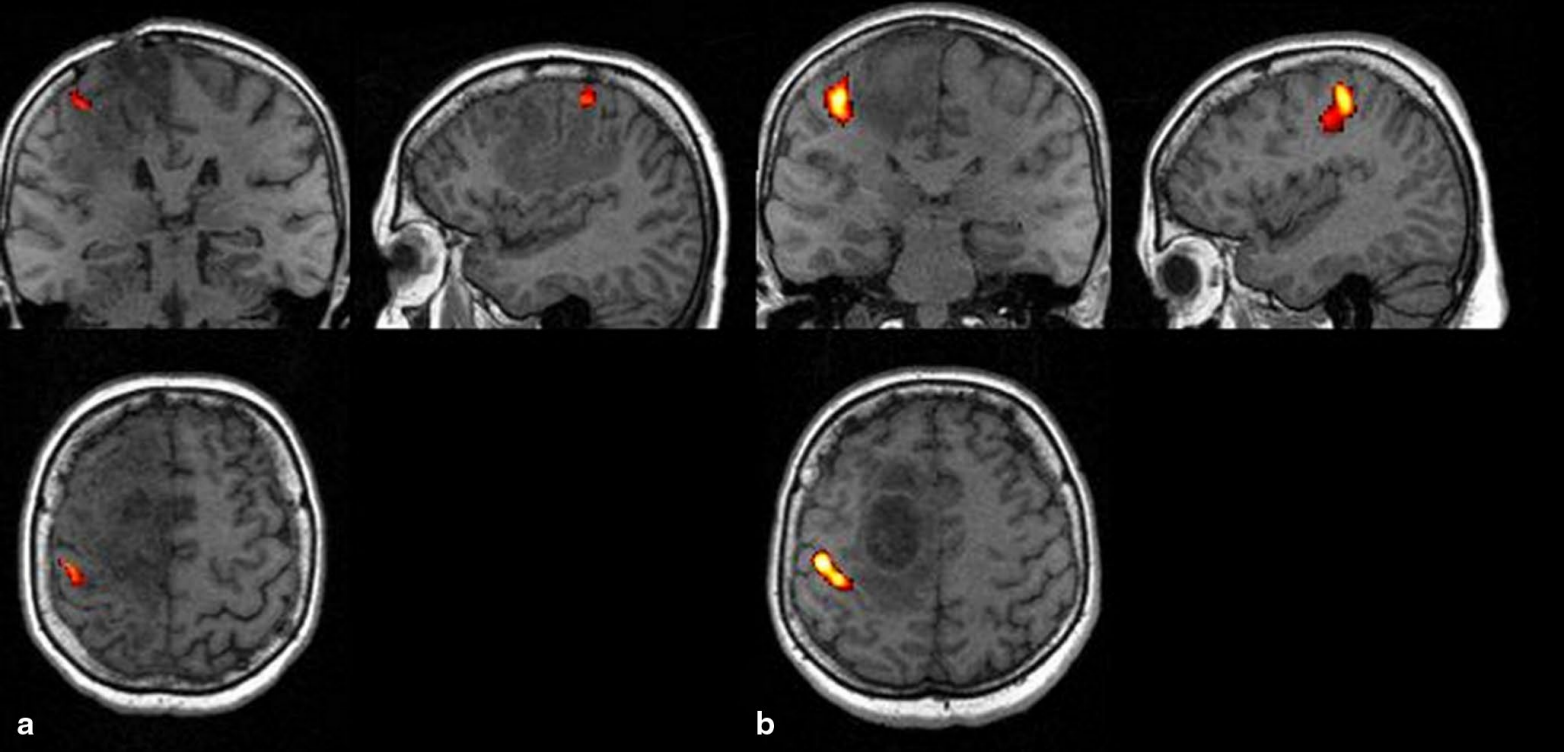
Patient JP. fMRI examinations, activation in primary motor area, (**a**) before, (**b**) after operation

**Fig. 2:**
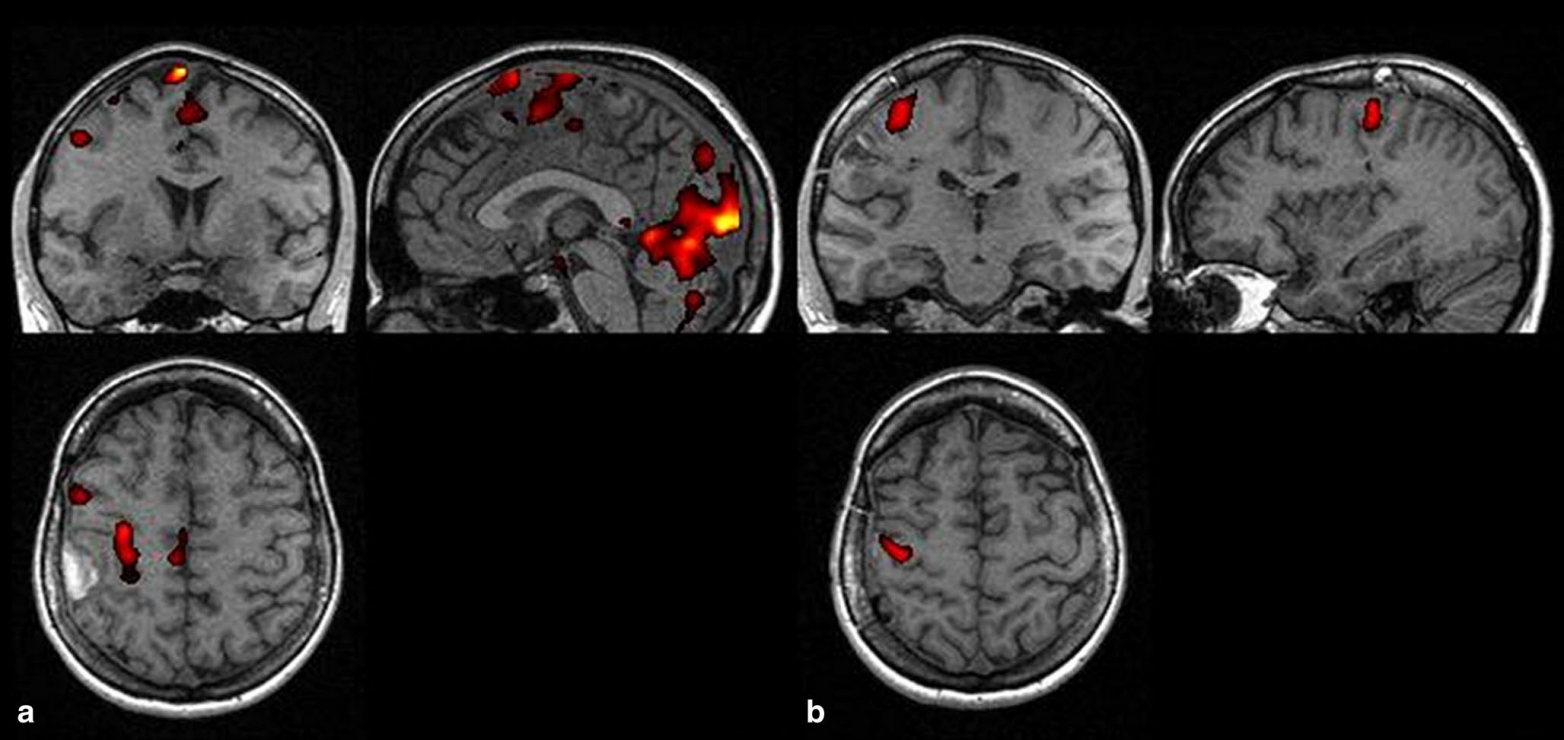
Patient MR. fMRI examinations, (**a**) activation in primary motor area and supplementary motor area before operation, (**b**) in primary motor area after operation

Following the surgery, the activation of M1a was documented in all the patients (*F* = 1.00). Aside from M1a, activation of SMAa (*F* = 0.78) and PMAa (*F* = 0.55) was also observed; however, activation of ipsilateral centers, M1u (*F* = 0.22) and PMAu (*F* = 0.11), was of rarer evidence. The activity of M1a was similar during the first and the second examination (0.94 vs. 1.0) (Fig. 1). Aside from M1a, the most frequently activated motor centers included:

- during the first examination: SMAa (0.75) followed by PMAa (0.50) (Fig. 2)
- during the second examination: SMAa (0.78) followed by PMAa (0.55).

In the case of ipsilateral centers, the frequency of activation during the second examination was lower than during the first examination.

**Fig. 3:**
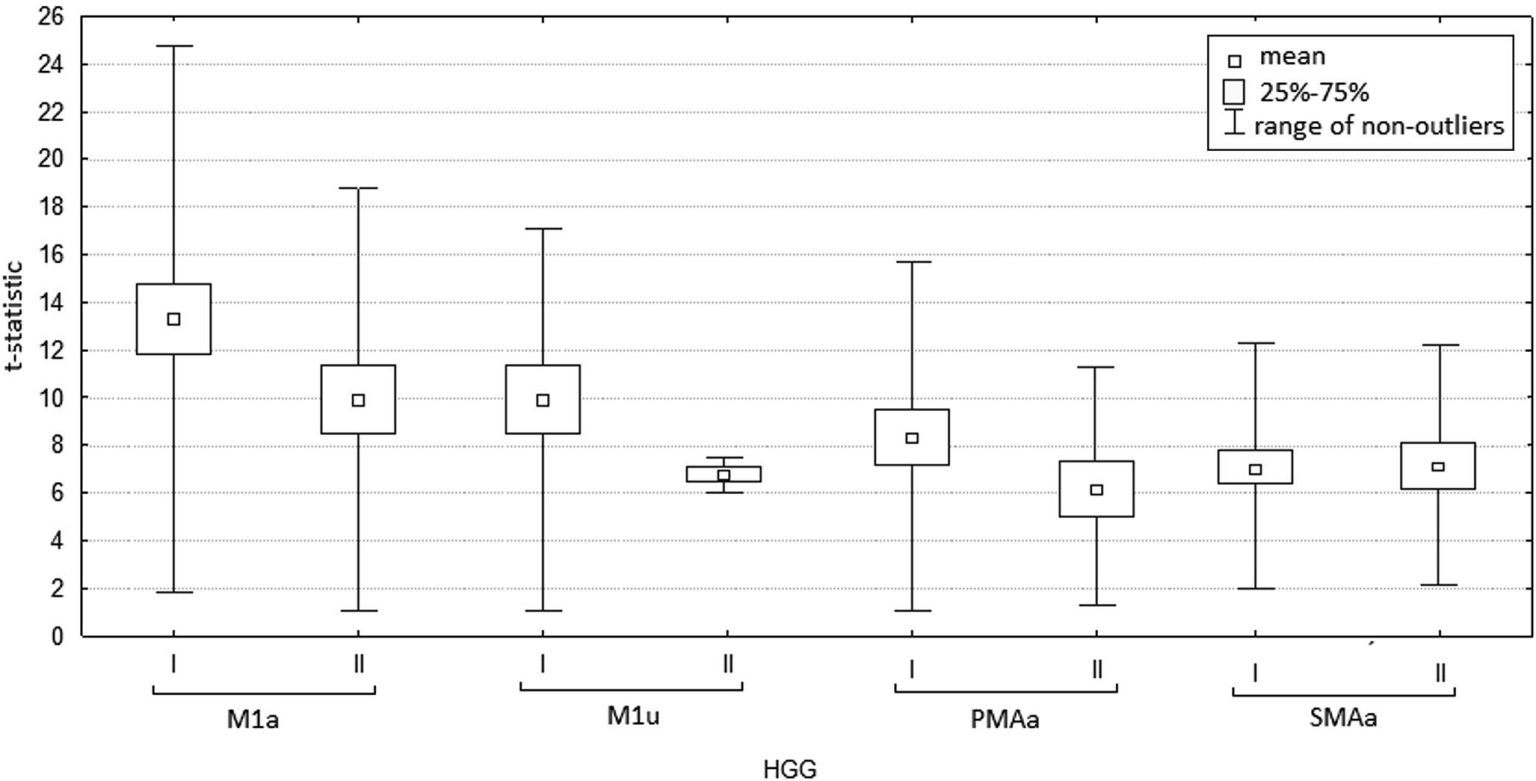
Values of t-statistics in analyzed motor areas in fMRI examinations. I—Before operation. II—After operation. *M1a* contralateral primary motor area, *M1u* ipsilateral primary motor cortex, *PMAa* contralateral premotor area, *SMAa* supplementary motor area

Mean values of t-statistics characterizing the intensity of activation during the first examination were higher than during the second examination; SMAa was the only center for which the mean values of activation intensity remained similar during both examinations (Fig. 3).

**Fig. 4:**
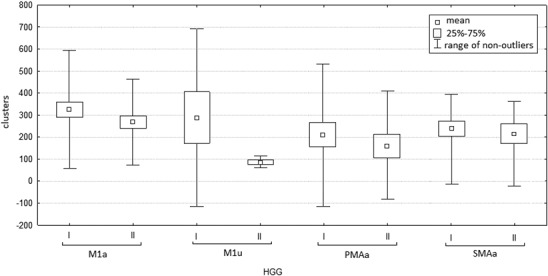
Number of clusters activated in analyzed motor areas during in fMRI examinations. I—Before operation. II—After operation. *M1a* contralateral primary motor area, *M1u* ipsilateral primary motor cortex, *PMAa* contralateral premotor area, *SMAa* supplementary motor area

During the second examination, the mean number of clusters activated in every analyzed motor area was lower than during the first examination (Table 3, Fig. 4).

**Table 3:** The comparison of frequency of activity (*F*), values of t-statistics (T) and number of clusters (*k*) of primary and secondary areas before (HGG^1^) and post-surgery (HGG^2^)

	HGG^1^ (*n* = 16)	HGG^2^ (*n* = 9)
M1a (*n*)	15	9
M1a (*F*)	0.94	1.0
T	13.32 ± 5.73	10.55 ± 4.20
*k*	326.0 ± 134.88	274.67 ± 102.88
M1u (*n*)	4	2
M1u (*F*)	0.25	0.22
T	7.84 ± 3.96	6.67 ± 0.36
*k*	222.0 ± 212.45	86.50 ± 13.44
PMAa (*n*)	8	5
PMAa (*F*)	0.50	0.55
T	8.34 ± 3.69	6.35 ± 2.56
*k*	210.13 ± 159.16	162.60 ± 122.20
PMAu (*n*)	4	1
PMAu (*F*)	0.25	0.11
T	7.05 ± 2.42	6.47
*k*	215 ± 108.9	178.0
SMAa (*n*)	12	7
SMAa (*F*)	0.75	0.78
T	7.15 ± 2.55	7.16 ± 2.51
*k*	240.92 ± 126.75	217.29 ± 122.52

*M1a* contralateral primary motor area, *M1u* ipsilateral primary motor cortex, *PMAa* contralateral premotor area, *PMAu* ipsilateral premotor area, *SMAa* supplementary motor area

No significant differences were observed between preoperative and postoperative frequency of activated areas, the intensity of the activation, and the mean number of activated clusters (*p* > 0.05).

## Discussion

HGGs represent nearly 80 % of all glial tumors. Glioblastoma multiforme, assigned to group IV according to WHO, is the most frequent histopathological type in this group, constituting the most frequent primary neuroepithelial tumor of the brain. Our study included solely the patients in whom the character of the tumor was confirmed histopathologically since the tumors of this group are considered distinct from other types of neuroepithelial tumors in terms of their biological characteristics. The aim of the project was to analyze the processes of brain plasticity for tumors characterized by the highest dynamics of proliferation process. Additionally, although it is considered to be extremely difficult in projects involving patients with brain tumors, we aimed to select the most homogenous study group. However, no single two cases, identical with regards to all the important parameters exist; the differences pertain to the size, shape, location (taking into account individual topography and cortical centers), internal microscopic structure, immunological characteristics, etc. [7, 8]. Therefore, we have decided to exclude patients with tumors classified up to histopathological grade III according to WHO classification.

The problem of inter- and intrapersonal variability of fMRI data analysis is well known [9]. To minimize misinterpretation two different radiologists identified the hot spots. In regards to the different mapping parameters as a source of mapping differences, the investigators strictly followed the mapping protocol.

Analysis of patients with HGG represents a challenge for functional fMRI examination, particularly with regards to the assessment of cortical motor centers. Usually, such patients show motor deficits due to the localization of pathological mass in the region of central sulcus. They experience problems with concentration, while even the shortest procedure of functional examination, lasting about 20–30 min, requires good cooperation of patient placed in an unfriendly environment of the MR scanner. The follow-up examination, scheduled and performed 3 months post-surgery, was challenging for the majority of our patients. As compared with the preoperative period, the clinical status of all the patients deteriorated due to a relatively short post-procedure time span, harmful effect of additional radio- and/or chemotherapy, and/or progression of the disease.

The second fMRI was conducted in 9 out of 16 patients. According to literature, such small group size is typical for fMRI studies of patients with central nervous system tumors [4, 10, 11]. Importantly, we did not find any published comparative studies of the results of preoperative and postoperative examination of HGG patients; this confirms the difficulties associated with such examination.

The second fMRI was conducted 3 months after the surgery. Such an interval was selected due to decreasing influence of such negative postoperative factors as swelling, the presence of hemorrhagic areas, and local disturbance of cerebral perfusion. Additional delay in the follow-up examination would probably be reflected by a further reduction in the number of patients eligible to the procedure of functional examination.

Individual results of our patients were variable with regards to the activation of various centers and the intensity and area of the activation. The results were modulated by the age and neurological status of patients, initial size of the tumor, its location, the size of surrounding swelling, the extent of resection, and postoperative outcome.

Overall, the results of fMRI examination in this group of patients are questionable. It is known that HGG can influence BOLD effect. Pathological mass and the associated swelling impair the local perfusion of the brain; furthermore, pathological vessels lack normal mechanisms of autoregulation. Potentially, these aforementioned factors can directly modulate BOLD effect, and therefore affect the result of fMRI. According to Schraiber et al., the activation of premotor cortex can be over-interpreted in the case of gliomas due to reduced BOLD effect in their proximity. Holodny et al. suggested that tumors with larger volumes can compress capillaries and small veins, which is reflected by weaker signal recorded in EPI sequence. In contrast, Schreiber et al. did not confirm the influence of tumor’s volume and mass effect on the distribution of cortical centers activity. According to these authors, the hypothetical compression of tumor on the surrounding vessels does not impair BOLD signaling or impairs it only to a minor extent [12–14]. A possible limitation of BOLD fMRI is radiation-induced neurovascular uncoupling, which can underestimate cortical activation [15]. The standard radiotherapy dose in HGGs is 40–50 Gy concentrated on tumor bed plus surrounding margin. From the oncological point of view none of the functional areas (regarding to natural history of infiltrating tumors) was sufficiently important to change the extent of radiotherapy area. In our study, we were not able to compare distance between the margin of radiotherapy field and the particular cortical centers.

Owing to not completely explained mechanism of BOLD effect, and the number and variety of its potential determinants, the results of fMRI studies should be interpreted with particular caution, particularly in the case of HGGs. Although fMRI should be considered as a helpful but inconclusive tool, it is still a unique method with increasing clinical application, enabling observation of the mechanisms of plasticity in human brain in vivo. Consequently, all observations, even general ones, are worthy of an analysis [10, 16].

The processes of motor cortex plasticity manifest by the activation of secondary cortical centers and the mobilization of the distal centers; these effects are not observed during the execution of simple movement by healthy volunteers [17–19]. In this study, the secondary centers, such as SMAa and PMAa, were the first to be activated in the presence of tumors with smaller volume; the activation of PMAu ipsilateral centers was of rarer evidence, and the activation of M1u was the rarest. The activation of ipsilateral hemisphere suggests that uncrossed pyramidal fibers gain an important role in the control and execution of movement. SMAa retained its frequency of activation in large-volume tumors, that is, those characterized by the proliferation of neoplastic mass; this area was followed by PMAa, and ipsilateral centers, M1u more frequently than PMAu. We also observed a relative increase in the frequency of activation in SMAa and M1u areas of large tumors as compared with low-volume lesions, along with the decreased frequency of activation of premotor cortex, both contralaterally and ipsilaterally (Table 2).

It is assumed that the processes of functional reorganization depend on patient’s age and the degree of disease dynamics, being most pronounced at a younger age and during chronic processes [1]. Most HGGs are diagnosed between 45 and 70 years of age, which is later than low-grade glioma, whose peak of incidence corresponds to the 3rd and 4th decade of life [20, 21]. Moreover, progression of HGG is extremely dynamic. Therefore, we can suspect the abovementioned factors to be responsible for determining a less radical change in the pattern of functional reorganization associated with larger volume of highly malignant tumors analyzed in this study.

Five of our patients were subjected to complete resection of the tumor; however, the majority of the subjects (*n* = 11) had undergone the subtotal resection. Therefore, the development of neoplastic disease and its natural progression were clearly not disturbed or reversed. Also, the surgery itself should be considered as constituting a harmful factor from the 3-month perspective [22]. The deterioration of the general clinical status and a higher degree of the upper limb paresis were observed in all the patients 3 months post-surgery (Table 1). Simultaneously, fMRI examination revealed lower intensity and reduced area of activation, despite unchanged frequency, in all but PMAu cortical centers. SMAa was the only center, which retained its activity. Despite the deterioration in neurological status, the preoperative and postoperative values of t-statistics of this center remained unchanged. During postoperative examination, contralateral premotor cortex was also confirmed as a center possessing an important role (Table 3).

On the basis of our findings, it can be concluded that SMAa plays the most important role in the processes of functional reorganization. It represents the most frequently activated area and its participation in the map of activation increases markedly both with the progression of disease and the proliferation of pathological mass; this was confirmed both in patients with higher volume of tumors and in individuals with deterioration in the general and neurological status. The existence of the abovementioned relationship was also documented by sparse reports from other authors [4, 23].

The frequency and intensity of PMAa activation ranks this center as second in the processes of plasticity. Consequently, it can be concluded that in patients with HGG the reorganization within the hemisphere containing pathological mass predominates. Perhaps, this phenomenon results from the higher number of active connections between the centers of the same hemisphere, promoting this pattern of plasticity, or from the local effect of pathological mass itself [24].

Despite existing controversies, an increasing number of authors have attributed longer survival of HGG patients, prolonged progression-free survival, and improved quality of life to a larger extent of resection [25, 26]. From clinical point of view, identification of the individual patterns of the reorganization of motor centers, and attempts toward protecting contralateral secondary centers can have high prognostic value with regards to the presence of motor deficits associated with surgical treatment [11].

## Conclusions

SMAa seems to play the most important role in the processes of motor cortex plasticity in HGG patients, both during the proliferation of the tumor, constituting the indicator of disease progression, and during the deterioration of neurological status due to other reasons.

Surgery seems not have a significant influence on the pattern of functional reorganization of the cortical centers for movement.

Identification of the individual patterns of the reorganization of motor centers plays an important role in clinical practice, both with regards to planning and performing surgical treatment.

### Conflict of Interest

On behalf of all authors, the corresponding author states that there is no conflict of interest.
